# A Surface Acoustic Wave-Based PM 1.0 Fine Dust Detection System Using Full Digital Time-Interleaved Counters

**DOI:** 10.3390/s24134149

**Published:** 2024-06-26

**Authors:** Chang-Hyeon Kim, Ki-Hoon Yang, Yeon-Seob Song, Sang-Sun Yoo, Younggun Pu, Il-Hwan Kim, Seok-Whan Chung, Kwang-Wook Choi, Jun-Eun Park, Kang-Yoon Lee

**Affiliations:** 1 Department of Electrical and Computer Engineering, Sungkyunkwan University, Suwon 16419, Republic of Korea; kch1036@skku.edu (C.-H.K.); yangghmail@skku.edu (K.-H.Y.); ssong0259@g.skku.edu (Y.-S.S.); syoo455@skku.edu (S.-S.Y.); hara1015@skku.edu (Y.P.); 2 Samsung Advanced Institute of Technology, Suwon 16706, Republic of Korea; ilhwan@samsung.com (I.-H.K.); swchung@samsung.com (S.-W.C.); k.-w.choi@samsung.com (K.-W.C.)

**Keywords:** time-interleaved, oscillator, RF amplifier, saw sensor, the mass of fine dust

## Abstract

This paper proposed a fine dust detection system using time-interleaved counters in which surface acoustic wave (SAW) sensors changed the resonance point characteristic. When fine dust was applied to the SAW sensor, the resonance point decreased. The SAW oscillator made of the SAW sensor and radio frequency (RF) amplifier generated an oscillation frequency that was the same as the resonance frequency. The oscillation frequency was transferred to digital data by a 20-bit asynchronous counter. This system has two channels: a sensing channel and a reference channel. Each channel has a SAW oscillator and a 20-bit asynchronous counter. The difference of the two channel counter results is the frequency difference. Through this, it is possible to know whether fine dust adheres to the SAW sensor. The proposed circuit achieved 0.95 ppm frequency resolution when it was operated at a frequency of 460 MHz. This circuit was implemented in a TSMC 130 nm CMOS process.

## 1. Introduction

Fine dust, composed of PM 2.5 and PM 1.0 ultrafine particles (UFPs), is a significant contributor to air pollution. It is linked to various health issues such as heart attacks and DNA mutations. Interest in developing equipment that can accurately measure the concentration of fine dust is increasing [1,2,3,4,5,6,7,8]. Various methods of measuring fine dust, including photoluminescence, filtration-based systems, crystal microbalance, light scattering and pulse count, taper device vibration microbalance, and weight measurement, are being studied [9,10,11,12,13].

Among these methods, the SAW sensor method has been proven to be more advantageous for fine dust detection than conventional sensors. The SAW sensor was developed using nanoparticle-based technology. It has a structure in which the presence of fine dust changes the resonance point.

**Figure 1:**
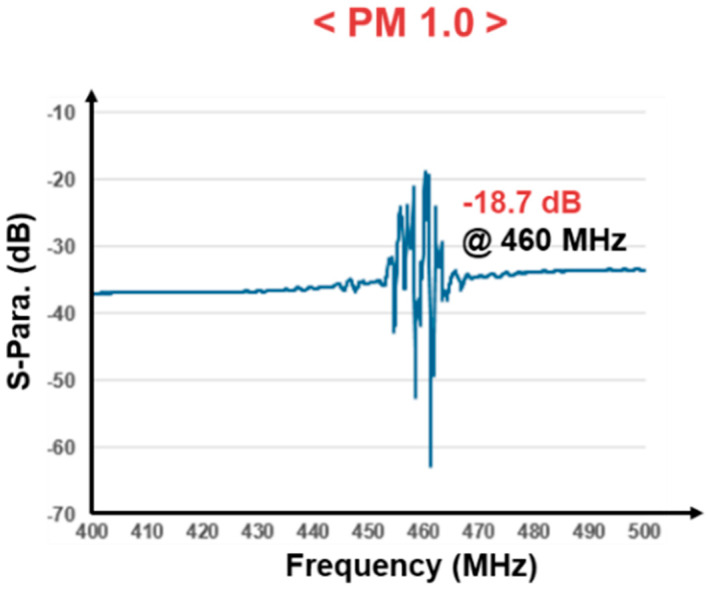
S-parameter (S21) of 460 MHz SAW sensor.

Figure 1 shows target SAW resonators, with one having a peak at 460 MHz. The average loss is about −35 dB, and the lowest loss is around −18.7 dB. For reliable oscillation using a feedback amplifier, the gain of the amplifier should be greater than 18.7 dB but less than 35 dB. If the gain exceeds 35 dB, the oscillation frequency will occur in the lower 460 MHz band. The oscillation frequency exhibits significant fluctuations. These fluctuations can introduce uncertainty and hinder the measurement of changes in the mass of fine dust. Therefore, the gain of the amplifier should be strictly controlled within an allowable range. As the resonance point can also be influenced by temperature and humidity, the method primarily involves using two sensors to detect changes solely caused by fine dust [14].

**Figure 2:**
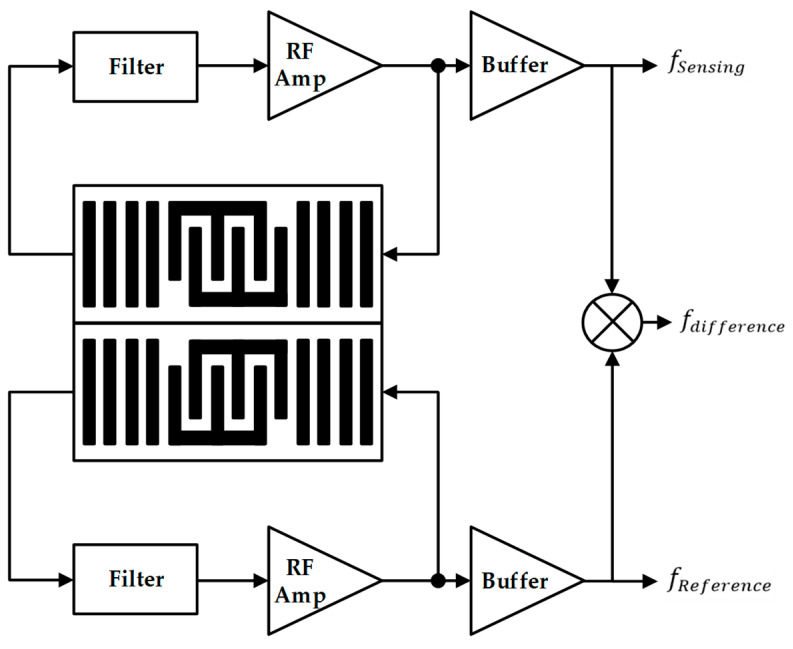
Block diagram of the prior frequency detecting system.

Figure 2 shows that two channels are composed in the existing frequency detection system. The difference in frequency generated in the two channels is checked using a mixer. One channel generates a frequency using a SAW sensor and an amplifier and checks the difference between the two frequencies using a mixer. Using characteristics of the SAW sensor, which changes the resonance point when fine dust is attached, the SAW sensor and amplifier change the frequency of the SAW oscillator. The frequency difference between the two channels means the presence or absence of fine dust. It can be visually confirmed [15,16,17,18,19,20,21].

However, there are several issues with how the frequency difference is assessed using the mixer. The smaller the frequency difference, the more susceptible it is to 1/f noise. Circuit improvement or a chopper filter is required to eliminate 1/f noise. Additionally, even with a frequency difference, a data processing circuit is necessary because the value must be examined through data processing. Another challenge is that the power consumption of the mixer used for frequency comparison is not lower than that of the amplifier. When the frequency increases, the power usage also increases. Furthermore, existing circuits are measured at the PCB level mainly utilizing SAW sensors and third-party IPs. Since various circuit configurations are needed to measure fine dust, the area required to measure the mass of fine dust is large.

## 2. The Proposed Architecture

**Figure 3:**
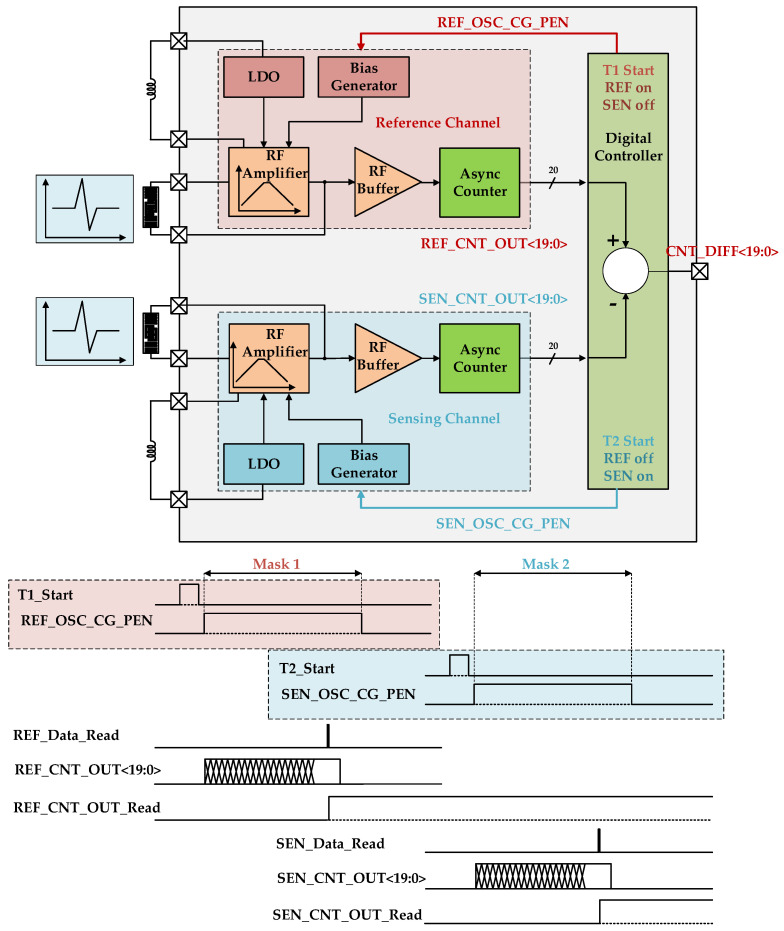
Block diagram (**above**) and timing diagram (**below**) of the frequency detection system.

Figure 3 shows a block diagram of the proposed system designed to detect changes in the mass of fine dust. As the mass of fine dust can influence the oscillator frequency in the system, both a reference channel and a sensing channel are essential. The sensor in the sensing channel induces a subtle change in the oscillation frequency based on the amount of fine dust. However, since the sensor is sealed, the oscillation frequency in the reference channel remains unaffected by the mass of dust.

By analyzing the difference in oscillation frequency between the reference channel and the sensing channel, the variation in the mass of fine dust can be measured and calculated. The oscillator consists of a SAW resonator and an amplifier in each channel. Given that the SAW resonator allows and passes through a specific frequency with very narrow resonance frequency, the feedback loop can only amplify the resonance frequency. Ultimately, if the loop gain exceeds the lowest loss of the SAW resonator, the loop will generate an oscillation frequency.

## 3. Building Blocks

### 3.1. RF Amplifier for SAW Oscillator

**Figure 4:**
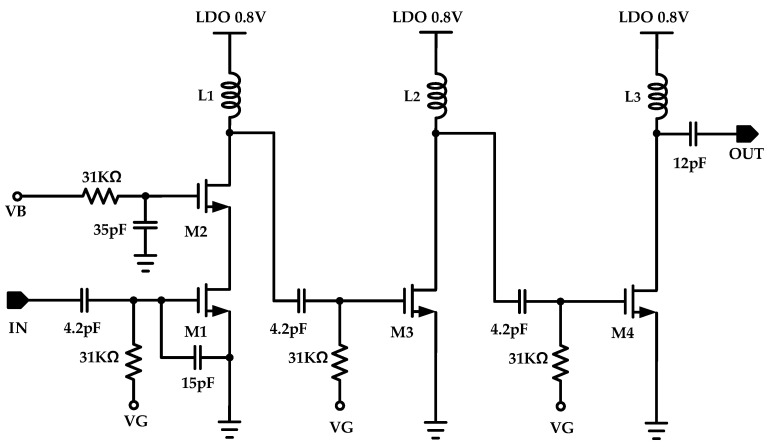
Block diagram of the 3-stage RF amplifier.

Figure 4 shows a schematic of the amplifier for oscillation using the SAW resonator. Since a single-stage amplifier lacks sufficient gain, exceeding 18.7 dB at 460 MHz, the amplifier is designed with three stages, considering a phase shift of −180°. To achieve a bandpass characteristic and controllable gain, switched capacitors and resistors were employed. Additionally, load inductors were implemented off-chip using high-Q inductors to reduce the chip area, as a 59 nH spiral inductor occupied a large chip area and increased costs.

A cascade amplifier offers advantages in terms of port isolation and high-frequency response, depending on the Miller capacitor. However, in the designed system, the cascade topology was adopted only for the 1st stage because the 2nd and 3rd stages required a large voltage headroom. Despite the considerably small voltage swing in the load of the 1st stage, the peak-to-peak voltage of the 2nd and 3rd stages swung from 0 V to twice VDD. Therefore, the 2nd and 3rd stages were designed with a single-stack amplifier having a large voltage headroom.

When we design an oscillator with the SAW resonator and amplifier, S-parameter analysis is useful for predicting the oscillation frequency with open-loop analysis. The open-loop gain of designed amplifier is defined as shown in Equation (1):

(1) $$1st\;stage\;Amplifier\;Gain=A_{v1}=g_{m1}(g_{m2}r_{o1}r_{o2}∥\omega L_{1}∥\frac{1}{\omega C})$$

(2) $$2nd\;and\;3rd\;stage\;Amplifier\;Gain=A_{v2,3}=g_{m3,4}(r_{o3,4}∥\omega L_{2,3}∥\frac{1}{\omega C})$$

(3) $$Open\;Loop\;Gain=A_{Loop}=A_{V1}A_{v2}A_{V3}$$

L1, 2, and 3 in Equations (1)–(3) are the inductance of the load inductor of each stage. C is designed based on the parasitic capacity value in order to minimize the value. The frequency band range was adjusted by adjusting the inductor according to the value of C. $g_{m1,2,3}$ is Siemens of M1, 2, 3, and 4.

The gain of the 1st stage was calculated to determine the impedance of the load inductor and capacitance in the cascode structure. It was calculated in parallel with the output resistance of the cascode structure. By calculating the gain of the 2nd and 3rd stages in the same way and adding them all together, the entire loop gain could be obtained.

**Figure 5:**
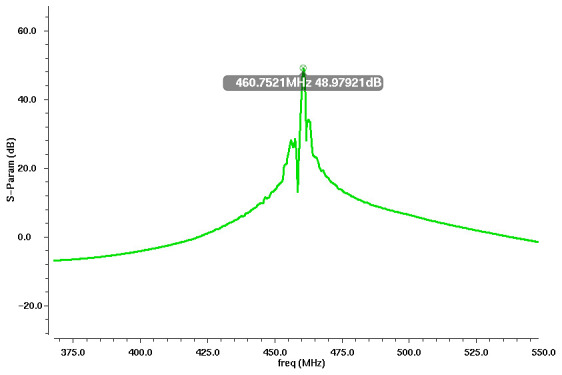
S21 simulation results of the 460 MHz SAW oscillator.

Figure 5 shows the simulation results of the loop gain for the 460 MHz SAW oscillator. To achieve maximum gain at 460 MHz, the loop gain involved modeling external components, incorporating a 59 nH inductor for both the 460 MHz SAW sensor and the RF amplifier.

**Figure 6:**
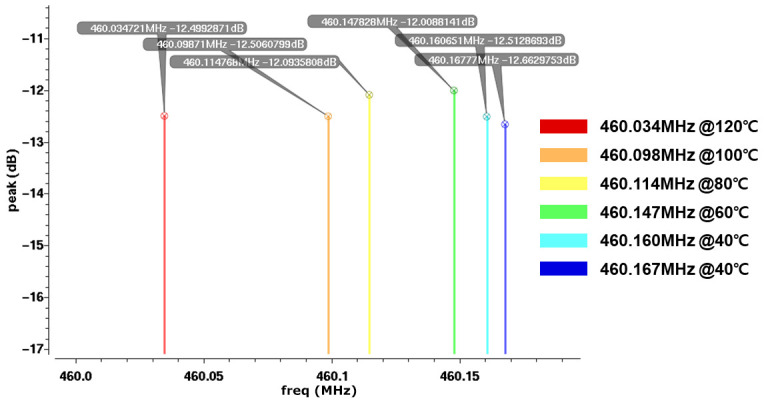
Periodic steady-state (PSS) simulation results of the 460 MHz SAW oscillator.

Figure 6 shows the results of periodic steady-state (PSS) simulation for a 460 MHz SAW oscillator. The oscillator generated using a 460 MHz SAW sensor and an RF amplifier exhibited vibration at the maximum gain point. This was confirmed by the PSS simulation results. In this process, the waveform and frequency could be observed at 460 MHz. From 20 to 120 Celsius, results were confirmed according to the temperature. The result that the frequency decreased with increasing temperature was confirmed.

### 3.2. The 20-bit Asynchronous Counter

To read the output frequency of the SAW oscillator, a 20-bit asynchronous counter was employed. The design aimed to achieve a resolution of less than 1 ppm for 460 MHz. To meet this requirement, a 20-bit or higher asynchronous counter was designed.

**Figure 7:**
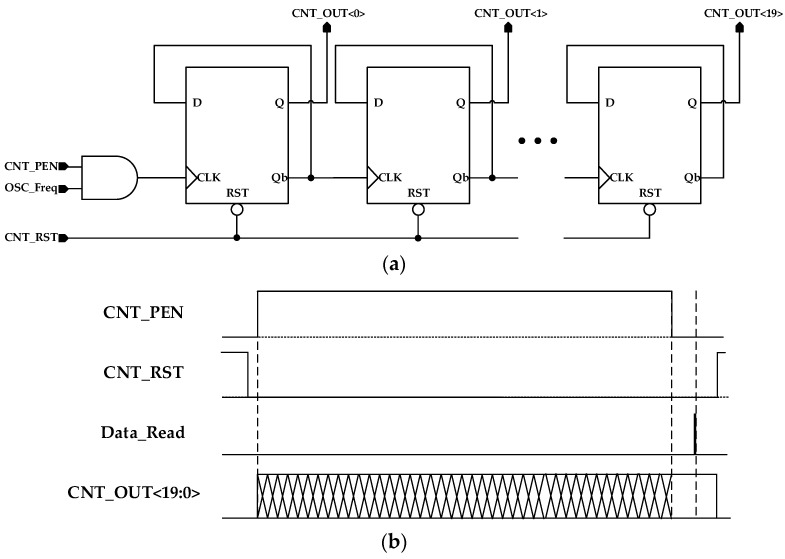
(**a**) Block diagram of the 20-bit asynchronous counter; (**b**) timing diagram of the 20-bit asynchronous counter.

Figure 7 illustrates the timing diagram of the 20-bit asynchronous counter. When CNT_PEN was high, the counter received the output of the SAW oscillator for counting. By defining the time when CNT_PEN is active as the “Mask”, the frequency can be determined using the mask length and the counter output:

(4) $$Oscillation\;Frequency=\frac{Counter\;output\left(DEC\right)}{Mask\;length}$$

The maximum mask length of the 20-bit asynchronous counter was determined by the number of bits. The maximum mask length was equal to $2^{bit}$ multiplied by the period of the oscillation frequency. Equation (4) shows that the frequency resolution corresponds to the change of one unit in the counter output. The frequency resolution is equal to 1/(mask length). For a maximum resolution of 0.95 ppm, it is possible to distinguish frequencies up to 460 MHz within a range of 438 Hz.

**Figure 8:**
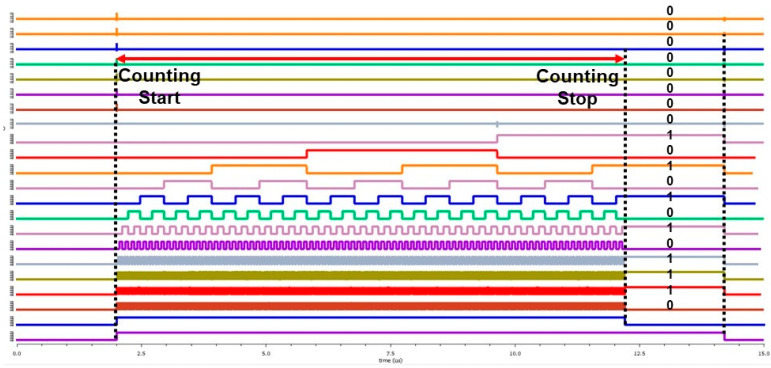
Transient simulation result of the 20-bit asynchronous counter.

Figure 8 shows the simulation results of a 20-bit asynchronous counter. It is the CNT_PEN signal that determines the counting start and stop. When CNT_PEN becomes high, counting starts. When CNT_PEN becomes low, counting stops, and the data are preserved. When CNT_PEN becomes low, the counting halts, and data are preserved. By utilizing the asynchronous counter, timing violations that may occur in 20-bit or higher counters are resolved. Furthermore, considering a delay between bit outputs, a margin is introduced to read the data after counting stops.

To compare frequencies received from the reference channel and sensing channel, a circuit was designed using a 20-bit asynchronous counter in this study. The power consumption of the mixer ranged from several hundred uW to several mW. Although this counter could operate at 3 GHz or higher with 20 bits, its power consumption remained below 100 uW. Additionally, it operated only once per second, with a maximum operational time of 6 ms, making it a negligible burden on battery consumption.

The two sensors can never be perfectly identical. Due to PVT variation in the ROIC, the oscillation frequencies of the two channels can never be the same. Therefore, an initial measurement was performed to carry out the baseline cancellation process in the counter. This involved subtracting an offset value from the counter to achieve the desired cancellation.

Simultaneously operating both channels revealed intermittent frequency discrepancies caused by interference between the sensing and reference channels due to the presence of the two sensors. When running a single channel independently, the precise measurement of frequencies for both channels was confirmed. However, when both channels were operated simultaneously, it was observed that their respective frequencies differed. This frequency variation was also observed when measuring with a spectrum analyzer and an oscilloscope.

To solve this problem, the time-interleave mode was activated. It was configured as shown in the block diagram and timing diagram of Figure 8. The reference channel was activated at T1, and the sensing channel was operated at T2. These two channels were operated separately according to the timing diagram. This improved pulling and the accuracy of the sensing process.

## 4. Measurement Results

**Figure 9:**
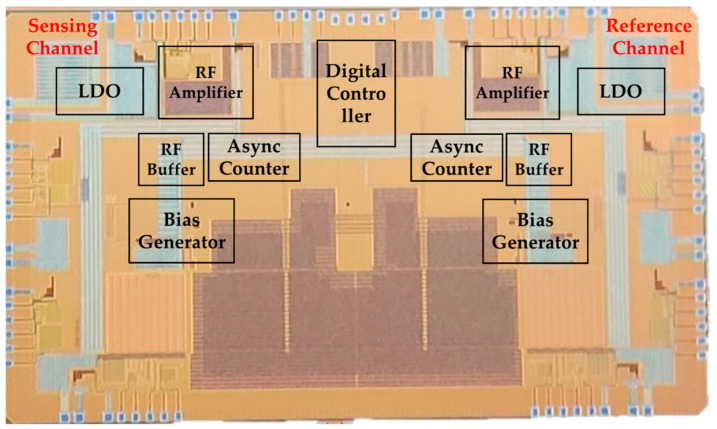
Chip photo of the fine dust mass measurement system.

The proposed sensor ROIC was designed using a 130 nm CMOS process. Figure 9 presents images of the chip and the board. Once designed, the ROIC was connected to the sensor to conduct oscillation testing. Off-chip inductors were used for matching. The use of a network analyzer confirmed an increase in the feedback loop’s gain, leading to the formation of the ROIC’s closed loop and resulting in oscillation. By converting counter values using Equation (4), it was observed that the oscillation frequency formed a peak.

**Figure 10:**
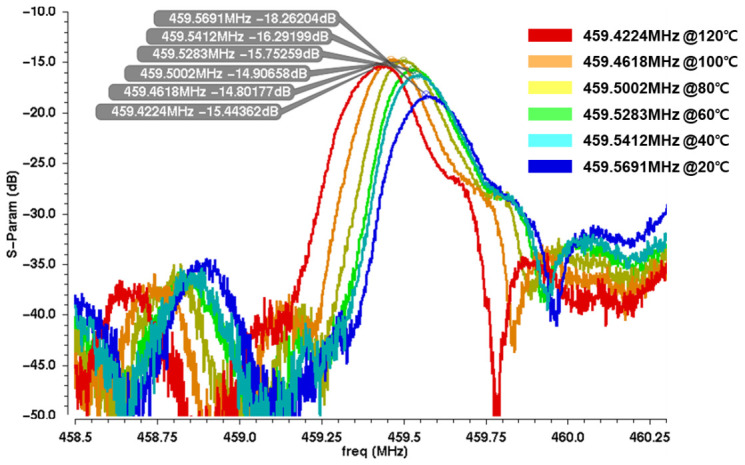
Frequency-shifting results by increasing the temperature of the SAW sensor.

Figure 10 shows the temperature-dependent S21 measurement results of the 460 MHz SAW sensor. Measurements were taken when the temperature varied from 0 to 120 °C using a chamber and sensor. Results were obtained at temperatures of 20, 40, 60, 80, 100 and 120 °C. As a result of extracting the S2P file by measuring it with a network animation and collecting it in simulation, the red graph shows the result at 120 °C, and the blue graph shows the result at 20 °C. Since the sensor’s characteristics can change with temperature, humidity, and fine particles with the pattern of change due to fine dust similar to that caused by temperature, a chamber was used. As the temperature increased, the resonance frequency decreased. Changes in the emission frequency were confirmed.

**Figure 11:**
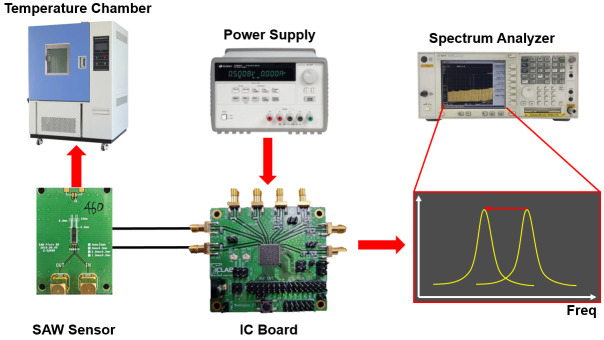
Measurement environment of the IC board for frequency shifting using a temperature chamber.

Figure 11 shows the measurement environment of the fine dust mass measurement system board. The sensor and the IC were connected to form an oscillator. The temperature was increased using a chamber. The oscillation frequency that changed during this process was checked using a spectrum analyzer.

**Figure 12:**
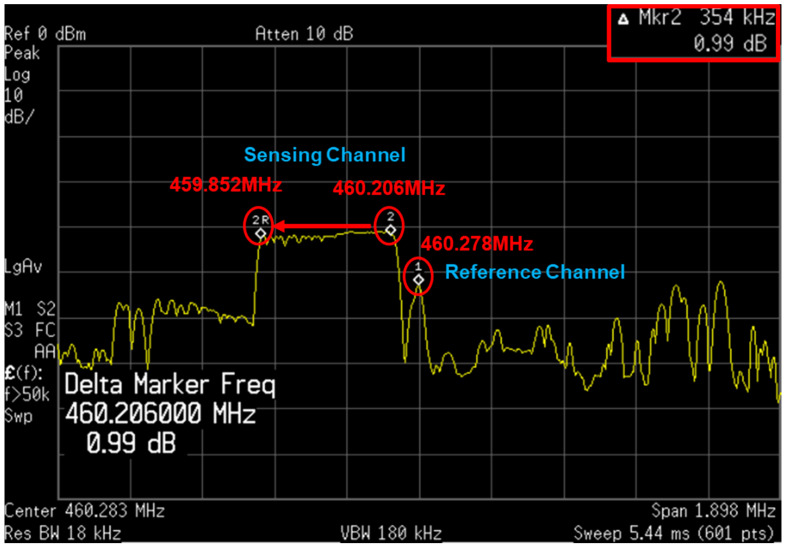
Frequency shifting results by rising temperature of the SAW sensor.

Figure 12 presents the results confirmed based on the data and the measurement environment from Figure 11. Changes in the emission frequency according to temperature variations were verified using a spectrum analyzer. It was confirmed that as the resonance point of the sensor changed, the emission frequency changed. This can be utilized to detect fine dust.

**Figure 13:**
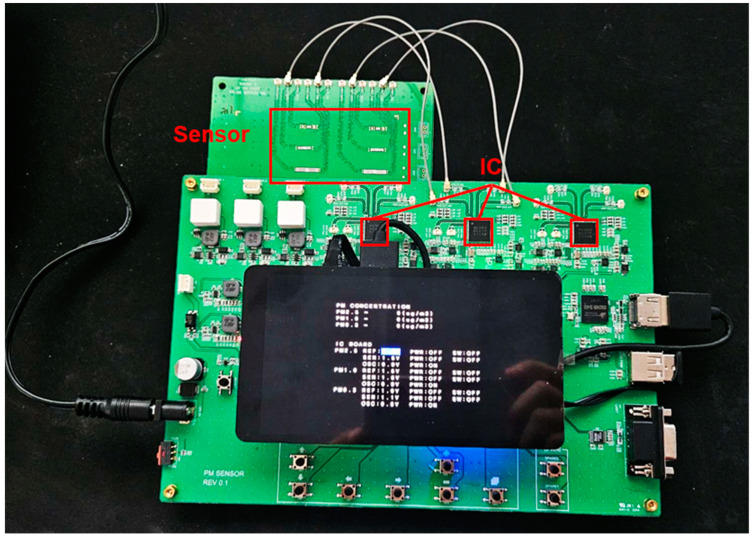
Fine dust mass measurement system board.

Figure 13 shows a photo of the fine dust mass measurement system board. We constructed an evaluation system board for fine dust mass measurement using six SAW sensors and three ICs. This system enabled the simultaneous detection of various types of fine dust particles. One type of fine dust might be detected by configuring one IC and two sensors in one set. The fine dust measurement system board was designed to target three types of fine dust. In the default state, the frequency difference between the reference channel and the sensing channel was measured.

**Figure 14:**
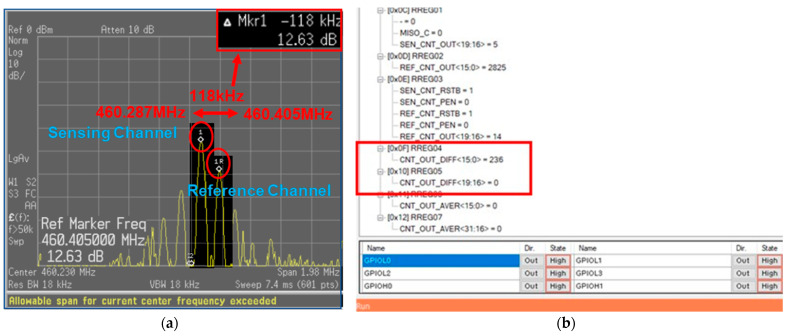
(**a**) Spectrum analyzer result of the sensing channel and reference channel; (**b**) counter output difference of the two channels.

Figure 14 shows the results of the frequency investigation of the two channels using the time-interleaved method. Results were obtained through both spectrum analyzer and counter reads. Figure 14a shows results of measurement through the spectrum analyzer. When checked with the spectrum analyzer, it was confirmed that the two frequencies normally output the desired frequency through the time-interleaved method. At this time, the two frequencies shows a difference of about 118 kHz. Figure 14b displays the output of the counter confirmed by SPI. The counter output is shown in decimal, and a result of 236 could be observed. The mask length was 2 ms, and a result of 118 kHz could be obtained by performing calculation using Equation (5) according to Equation (4).

(5) $$118\;kHz(Oscillation\;Frequency)=\frac{236(Counter\;output)}{2\;ms(Mask\;length)}$$

As mentioned in Section 4, when connecting and testing two sensors, we observed a phenomenon where the oscillation frequencies were pulling together. By activating the time-interleaving feature, we confirmed that the performance was normal. Although implementing the actual measurement environment was challenging, we were able to accurately measure the frequency variation between the reference and sensing channels due to environmental changes, although we could not directly inject fine dust for measurement. The total power consumption of the entire system measured during the test was 12 mW. Since each channel operated independently, we calculated the power consumption value when a single channel was active. The board was configured to accommodate temperature measurements using connected cables. However, in practical use, a sensor can be inserted, and the size is minimized accordingly.

Table 1 below summarizes the performances of previously published ROICs for fine dust sensors. An objective performance comparison might not hold significant meaning due to there being various design approaches depending on the sensor capabilities. Nevertheless, it could be observed that compared to other papers, the proposed ROIC exhibited superior or comparable performance [22].

The data in Table 1 show that the frequency comparison circuit consumes less power than the conventional circuit. Since the results of comparing frequencies consisted of digital data, data processing was easy. The entire circuit was designed to be on-chip. The target frequency was around 460 MHz. The results are all written according to the target. Since the goal of the resolution was less than 1 ppm, it satisfied 0.95 ppm. If the output of the asynchronous counter is increased by more than 20 bits, the resolution can be increased.

In addition, compared to other reference circuits, our circuit could oscillate a wide range of frequencies. It was designed to have sufficient gain as a target from (200 MHz to 2 GHz). This allowed sufficient gains to be obtained in a wide frequency range because the gain requirement of the amplifier varied, depending on the performance of the sensor.

## 5. Conclusions

In this paper, we fabricated and validated an ROIC for fine dust measurement sensors. Unlike the conventional structure using mixers, we utilized digital counters for calibration, enhancing the sensitivity of sensing by taking advantage of its robustness against external noise. To address the issue of frequency pulling and increased sensing error when operating both the reference and sensing channels simultaneously, we implemented a time-interleaving technique on the board and applied it to the counter. This allowed the problem to be overcome. It also allowed the desired performance of the ROIC module to be achieved. Considering that implementing the time-interleaving technique in the IC would not increase the board size, we expect to develop an excellent ROIC without size constraints.

## Figures and Tables

**Table 1:** Summary of performance comparison.

	This Work	Ref. [5]	Ref. [7]	Ref. [8]	Ref. [11]
Frequency (MHz)	460	60	433.9, 468	1396	400–1000
Architecture (Calculation of Frequency)	Counter	Mixer	Mixer	Mixer	N/A
Power Consumption of Comparison Block (mW)	0.11	130	-	1.6	0.4 (1-Mixer)
Resolution (ppm)	0.95	0.4512	53	-	-
Supply Voltage (V)	1.5	5	-	1.8	1.2
Process (nm)	130	Off-Chip	Off-Chip	180	65

## Data Availability

Data is contained within the article.
